# Succinate coenzyme A ligase β‐like protein from *Trichinella spiralis* is a potential therapeutic molecule for allergic asthma

**DOI:** 10.1002/iid3.1321

**Published:** 2024-06-18

**Authors:** Kalibixiati Aimulajiang, Wen Chu, Shuyi Liao, Zhaohai Wen, Rongdong He, Mingmin Lu, Lixin Xu, Xiaokai Song, Xiangrui Li, Ruofeng Yan

**Affiliations:** ^1^ Laboratory of Animal Health and Food Safety, MOE Joint International Research, College of Veterinary Medicine Nanjing Agricultural University Nanjing Jiangsu P. R. China; ^2^ State Key Laboratory of Pathogenesis, Prevention and Treatment of High Incidence Diseases in Central Asia, Clinical Medicine Institute The First Affiliated Hospital of Xinjiang Medical University Urumqi Xinjiang P. R. China; ^3^ Changsha Animal and Plant Disease Control Center Changsha Hunan P. R. China; ^4^ Department of Veterinary Medicine, College of Coastal Agricultural Sciences Guangdong Ocean University Zhanjiang P. R. China

**Keywords:** allergic asthma, helminth molecules, immune regulation, Succinate Coenzyme A ligase beta‐like protein, *Trichinella spiralis*

## Abstract

**Background:**

For decades, studies have demonstrated the anti‐inflammatory potential of proteins secreted by helminths in allergies and asthma. Previous studies have demonstrated the immunomodulatory capabilities of Succinate Coenzyme A ligase beta‐like protein (SUCLA‐β) derived from *Trichinella spiralis*, a crucial excretory product of this parasite.

**Objective:**

To explore the therapeutic potential of SUCLA‐β in alleviating and controlling ovalbumin (OVA)‐induced allergic asthma, as well as its influence on host immune modulation.

**Methods:**

In this research, we utilized the rTs‐SUCLA‐β protein derived from *T. spiralis* to investigate its potential in mitigating airway inflammation in a murine model of asthma induced by OVA sensitization/stimulation, both pre‐ and post‐challenge. The treatment's efficacy was assessed by quantifying the extent of inflammation in the lungs.

**Results:**

Treatment with rTs‐SUCLA‐β demonstrated efficacy in ameliorating OVA‐induced airway inflammation, as evidenced by a reduction in eosinophil infiltration, levels of OVA‐specific Immunoglobulin E, interferon‐γ, interleukin (IL)‐9, and IL‐17A, along with an elevation in IL‐10. The equilibrium between Th17 and Treg cells plays a pivotal role in modulating the abundance of inflammatory cells within the organism, thereby ameliorating inflammation and alleviating symptoms associated with allergic asthma.

**Conclusions and Clinical Relevance:**

Our data revealed that *T. spiralis*‐derived Ts‐SUCLA‐β protein may inhibit the allergic airway inflammation by regulating host immune responses.

## Graphical abstract

rTS‐Succinate Coenzyme A ligase beta (SUCLA‐β) can reduce the levels of eosinophils around bronchus and blood vessels and the number of inflammatory cells in bronchoalveolar lavage fluid. rTS‐SUCLA‐β could reduce the Immunoglobulin level of the mouse upon OVA sensitization/challenge. rTS‐SUCLA‐β treatment regulates host immunity by promoting Treg cells differentiation and inhibiting Th17 cells differentiation and plays an important role in allergic asthma.

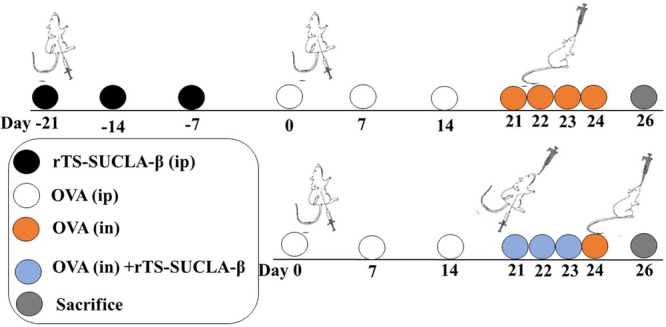

## 1 INTRODUCTION

Allergic asthma is often defined as asthma associated with sensitization to air allergens, characterized by recurrent wheezing and bronchial constriction, and sensitization to air allergens is an important cause of asthma symptoms and airway inflammation.^1^ Asthma impacts a global population of 300 million individuals and results in a minimum of 250,000 fatalities annually.^2^ Despite adherence to maximally optimized medication and treatment of associated factors, approximately 6% of patients with asthma continue to have severe asthma, resulting in a reduced quality of life and an increased risk of hospitalization and death.^3^, ^4^ As suggested by the hygiene hypothesis, at present, many studies have found that asthma infection rates are lower in areas where parasites are endemic.^5^ Epidemiological studies have confirmed the role of helminth infections (such as hookworm) in reducing the risk of asthma, but not all parasites protect against asthma.^6^, ^7^

Th2 immune response, which induces the secretion of IL‐4, IL‐5, and IL‐13 by both innate and adaptive immune cells, is the main pathway of most parasitic immunity and immunopathological processes in asthma.^8^ Furthermore, the presence of Th1 and Th17 cell immune responses has been linked to both parasitic infections and the severity of asthma symptoms.^9^, ^10^ Previous studies have found that the excretory/secretory products (ESPs) of *Fasciola hepatica* can inhibit the accumulation of mucus, eosinophils, and lymphocytes in the airways of allergen‐challenged mice.^11^ Somatic products of *Marshallagia marshalli* were able to suppress the induction of allergic airway inflammation in mice.^12^ *Schistosoma mansoni* recombinant proteins (Sm22.6 and Sm29) or antigens derived from *schistosoma* tegument regulate IL‐5 and IL‐13 production and enhance eosinophil production in asthma models.^13^

Upon ingestion by a susceptible host, the muscle larvae (ML) of *T. spiralis* infiltrate the columnar cells within the epithelium of the small intestine.^14^ Subsequently, they undergo four molts and mature into adults, engaging in reproductive activities to generate new larvae that enter the bloodstream via lymphatic vessels.^15^, ^16^ These larvae circulate through the bloodstream until they reach striated muscle tissue, where they invade muscle cells and commence an extracellular existence.^15^, ^16^ After a period of 20 days elapses, these larvae become infectious and can initiate a new life cycle upon being ingested by another host.^15^, ^16^ Previous studies have shown that the therapeutic effect of *T. spiralis* ESPs on inflammatory colitis is achieved by inducing regulatory T cells.^17^ In a mouse model of asthma induced by ovalbumin (OVA), soluble products from *T. spiralis* adults improved airway inflammatory responses.^18^

The *T. spiralis* ML ESPs protein Succinate Coenzyme A ligase beta‐like protein (SUCLA‐β), which belongs to the succinate‐CoA synthetase gene, plays a role in the citric acid cycle.^19^ Our previous study found that rTs‐SUCLA‐β can bind to rat peripheral blood mononuclear cells (PBMCs) and significantly inhibit the proliferation rate, migration, and monocyte phagocytosis of PBMCs.^20^ In vivo, inhibition of IL‐17A secretion and a significant increase in levels of anti‐rTs‐SUCLA‐β immunoglobulin (IgG, IgG1, and IgG2a) promoted a significant reduction in ML of *T. spiralis*.^20^ The findings indicate that rTs‐SUCLA‐β has the potential to serve as a promising candidate for managing *T. spiralis* infection through its ability to suppress immune cell activity and decrease parasite load. Moreover, it could potentially be utilized in the treatment of autoimmune disorders and prevention of organ transplant rejection by inhibiting the host's IL‐17A immune response.

The objective of this research was to examine the diverse immunomodulatory impacts of *T. spiralis* SUCLA‐β on mice with OVA‐induced allergic asthma and its influence on the secretion of cytokines. This study aims to identify promising worm molecules for future treatment strategies targeting allergic and autoimmune disorders.

## 2 METHODS

### 2.1 Animals

Female Balb/c mice aged 6‐8 weeks were obtained from Yangzhou University and housed in a sanitized facility at Nanjing Agricultural University, where standardized protocols were followed. The animal study was conducted in compliance with the guidelines of the Animal Ethics Committee (approval number: PZ2019013) at Nanjing Agricultural University.

### 2.2 The production and isolation of rTS‐SUCLA‐β protein

Recombinant plasmid expression TS‐SUCLA‐β (pET‐32a (+)/TS‐SUCLA‐β; GenBank: Tsp_03823) was preserved by this research laboratory. *Escherichia coli* BL21 (Vazyme Nanjing) transformed with pET‐32a (+)/TS‐SUCLA‐β was induced, and the rTS‐SUCLA‐β was purified as previously described.^20^ The protein labeled with histidine in pET‐32a was also prepared using the identical method mentioned earlier, serving as a control protein for subsequent functional analysis. A ToxinEraser^TM^ Endotoxin Removal Kit (GenScript) was employed to eliminate lipopolysaccharide from the Ts‐SUCLA‐β protein. The endotoxin level of the Ts‐SUCLA‐β protein was assessed using the Chromogenic LAL Endotoxin Assay Kit (Beyotime), yielding a value below 0.1 EU/mL.

### 2.3 OVA sensitization and treatment

**Figure 1:**
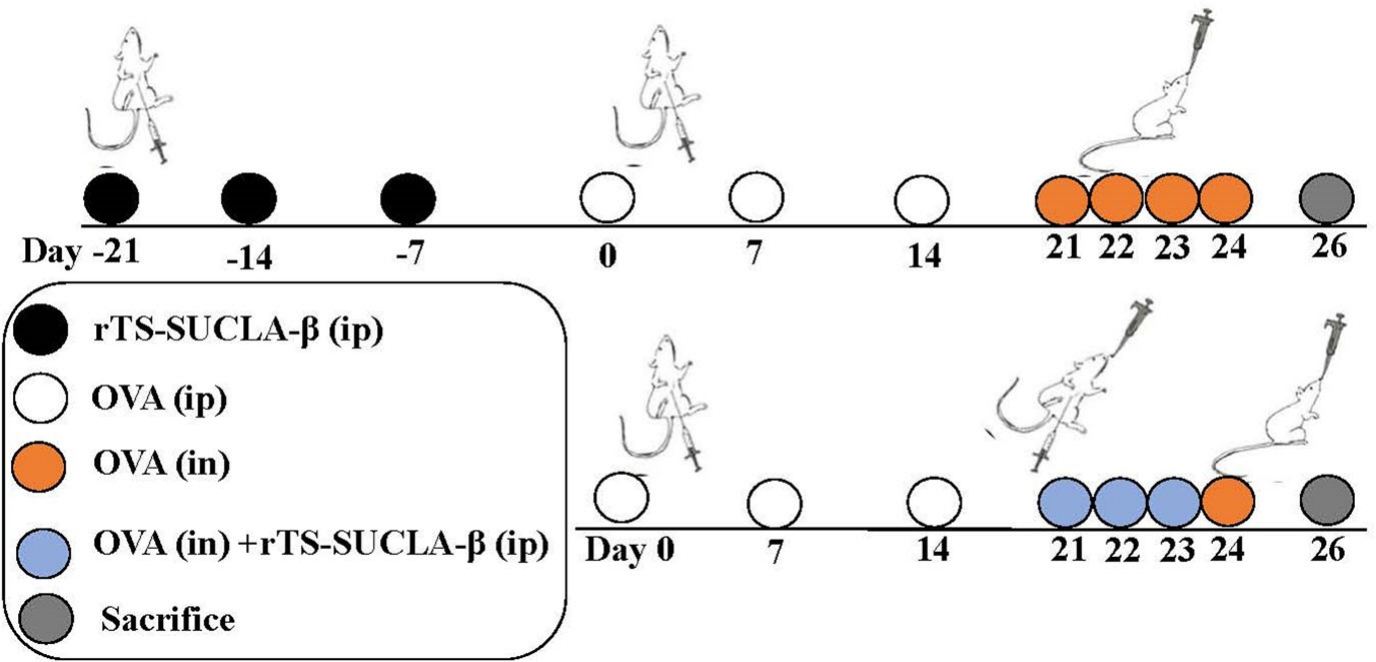
Regimen for mouse OVA sensitization and rTS‐SUCLA‐β treatment.

Female Balb/c mice were randomly allocated into 10 experimental groups. Phosphate buffered saline (PBS) control group, OVA group, treatment group (OVA + 250rTS‐SUCLA‐β, OVA + 50rTS‐SUCLA‐β, OVA + 10rTS‐SUCLA‐β, and OVA+pET‐32a), prevention group (250rTS‐SUCLA‐β + OVA, 50rTS‐SUCLA‐β + OVA, 10rTS‐SUCLA‐β + OVA and pET‐32a + OVA). In each group, Balb/c mice were sensitized intraperitoneally (ip) with a total volume of 200 μL containing 100 μg of OVA (Sigma‐Aldrich) and 20 μg of Imject Alum (Pierce, Sigma‐ Aldrich), on Day 0, 7 and 14. Following 1 week after the final sensitization, all mice received intranasal challenges (in) under sedation using chloral hydrate (8 μg/20 g). The challenges consisted of a total volume of 50 μL sterile PBS containing 100 μg of OVA dissolved in it for four consecutive days on Day 21, 22, 23, and 24. In the preventive groups, mice were administered different doses (250, 50, and 10 μg) of rTS‐SUCLA‐β or 50 μg of pET‐32a protein via intraperitoneal injection before OVA sensitization on days −21, −14, and −7. Receiving therapeutic treatment, mice were intraperitoneally co‐administered with varying doses (250, 50, and 10 μg) of rTS‐SUCLA‐β or 50 μg of pET‐32a protein while undergoing OVA sensitization on days 21, 22, and 23. The control group for comparison consisted of mice from the PBS group, which did not undergo any intervention or challenge (Figure 1). After the completion of the final challenge, a humane method was employed to euthanize all mice within 48 h. Lung tissues were then gathered for the purpose of evaluating inflammation. Furthermore, subsequent immunological analyses involved obtaining bronchoalveolar lavage fluid (BALF), spleen cells, and sera samples.

### 2.4 Receiving mouse serum and BALF samples

Forty‐8 h elapsed following the final encounter with OVA, five mice from each group underwent orbital blood sampling and were subsequently euthanized. The collected mouse blood was incubated at 37°C for 1 h, then overnight at 4°C, centrifuged at 1800×g for 10 min, and the upper serum was collected and stored at −80°C. After euthanizing the mice, the lungs were subjected to tracheal lavage using 0.8 ml of sterile PBS for three consecutive washes. The BALF obtained from each mouse was subjected to centrifugation at 1800×g for 10 min at a temperature of 4°C. Subsequently, the resulting supernatants were carefully preserved at −80°C for storage purposes. Cells present in the precipitate were resuspended in 0.5 ml of PBS and subsequently subjected to Wright's and Giemsa staining (Solarbio, Beijing, China) on glass slides. Subsequently, macrophages, lymphocytes, neutrophils, and eosinophils were quantified using a light microscope (Olympus IX71, Tokyo, Japan).

### 2.5 Measurement of cytokines in BALF

ELISA kits (Enzyme‐linked Biotechnology, Shanghai, China) were utilized to measure the levels of interferon (IFN)‐γ, IL‐4, IL‐10, IL‐17A and IL‐9 in BALF according to the manufacturer's instructions. The optical density was assessed at 450 nm (OD450) using an ELISA plate reader (Thermo Fischer Scientific, Waltham, MA, US).

### 2.6 Isolation of spleen lymphocytes and histopathology analysis of pulmonary

Forty‐8 h after the last challenge with OVA, another 5 mice in each group received blood from orbit and were killed, and the mice were exposed to 75% ethanol. The mice soaked in 75% ethanol were taken into the ultra clean table, and the spleen and lung were removed by opening the abdomen. Spleen lymphocytes were isolated according to the instructions of splenic lymphocyte separation solution (Solarbio, Beijing, China) and diluted into cell suspension of 1×10^6 ^mL in cell culture. The lung tissues were fixed using formalin and subsequently embedded in paraffin. Following this, sections were meticulously cut and subjected to staining with hematoxylin/eosin (H&E) (Solarbio, Beijing, China), as per established protocols. The degree of cell infiltration around the basal membrane of bronchi or vessels was microscopically assessed to determine the inflammation of lung tissues, graded on a scale ranging from 0 to 3. A score of 0 indicated occasional infiltration or absence of inflammatory cells in lung tissue, while a score of 1 denoted the presence of a thick layer consisting of two to three inflammatory cells surrounding bronchi or vessels. Similarly, a score of 2 represented the presence of four to five inflammatory cells forming a thick layer around bronchi or vessels, and finally, a score of 3 indicated the presence of more than five inflammatory cells encompassing bronchi or vessels.^21^

### 2.7 Detection of total Immunoglobulin (IgE) and OVA‑specific IgE in sera

The ELISA kits from Enzyme‐linked Biotechnology in Shanghai, China were utilized to quantify the concentrations of total IgE and OVA‐specific IgE in mouse sera. The optical density was measured at 450 nm (OD450) using an ELISA plate reader from Thermo Fischer Scientific in Waltham, MA, US according to standard protocols provided by the manufacturers.

### 2.8 Intracellular cytokine staining and flow cytometry

The expression of markers on T helper cells, isolated from mouse spleen lymphocytes and stimulated with Leukocyte Activation Cocktail (BD Pharmingen), was assessed using flow cytometry analysis.^22^ Before performing intracellular staining, the Leukocyte Activation Cocktail (BD Pharmingen) was introduced into a 24‐well plate filled with RPMI 1640 medium. The plate was then incubated at a temperature of 37°C in an environment containing 5% CO2 for a duration ranging from 4 to 6 h. Afterwards, the cells were moved and subjected to centrifugation at 500 rcf for a period of 10 min. Subsequently, they underwent three washes using fluorescence‐activated cell sorting (FACS) stain buffer (BD Pharmingen) and were then stained with mouse‐specific antibodies that were conjugated with fluorescein‐5‐isothiocyanate, P‐phycoerythrin (PE), antigen presenting cells (APC), and PE‐Cy5.5. This staining process lasted for approximately 40 min at a temperature of 4°C. The mouse antibodies against CD4, CD25, IL‐17A, and FOXP3 were acquired from BD Biosciences (Franklin Lakes, USA). After subjecting the cells to centrifugation at a speed of 500 rcf for 5 min at a temperature of 4°C, we introduced 1 ml of Fix/Perm Buffer (BD Biosciences, USA) and allowed it to incubate in a light‐restricted environment for a duration of 20 min. The cells were thoroughly washed and subsequently resuspended in 350 μL of stain buffer. The FACS Canto II flow cytometer (Becton Dickinson) was utilized for data acquisition, with gating established at a cell count of 100,000. To evaluate the expression of Treg and Th17 cells, intracellular cytokine antibodies for IL‐17A and FOXP3 were employed for cell staining.

### 2.9 Statistical analysis

The information in this research was acquired from three separate trials and presented as the average ± SEM (standard error of the mean). Statistical examination was performed, utilizing one‐way analysis of variance to detect any disparities within groups before conducting comparative investigations. A p‐value lower than 0.05 was deemed statistically significant.

## 3 RESULT

### 3.1 Expression and purification of rTS‐SUCLA‐β

**Figure 2:**
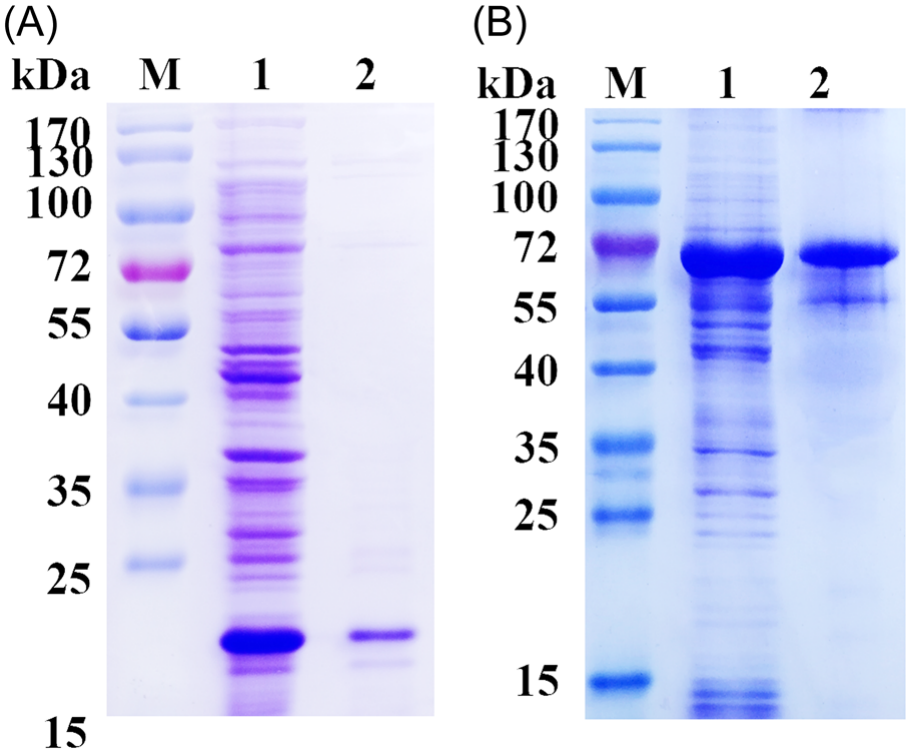
(A): Expression and purification of pET‐32a protein; Lane M: Standard protein molecular weight Marker, Lane 1: Before purification of pET‐32a protein, Lane 2: Purified pET‐32a protein. (B): Expression and purification of rTS‐SUCLA‐β; Lane M: Standard protein molecular weight Marker, Lane 1: Before purification of rTS‐SUCLA‐β, Lane 2: Purified rTS‐SUCLA‐β.

In sodium dodecyl‐sulfate polyacrylamide gel electrophoresisof recombinant TS‐SUCLA‐β protein a band of approximately 47 kDa was detected, and this measurement remained consistent even after reducing the size of the His‐tagged fusion protein derived from the pET‐32a vector (18 kDa) (Figure 2).

### 3.2 Reduced allergic asthma in lungs of mice treated with rTS‐SUCLA‐β

**Figure 3:**
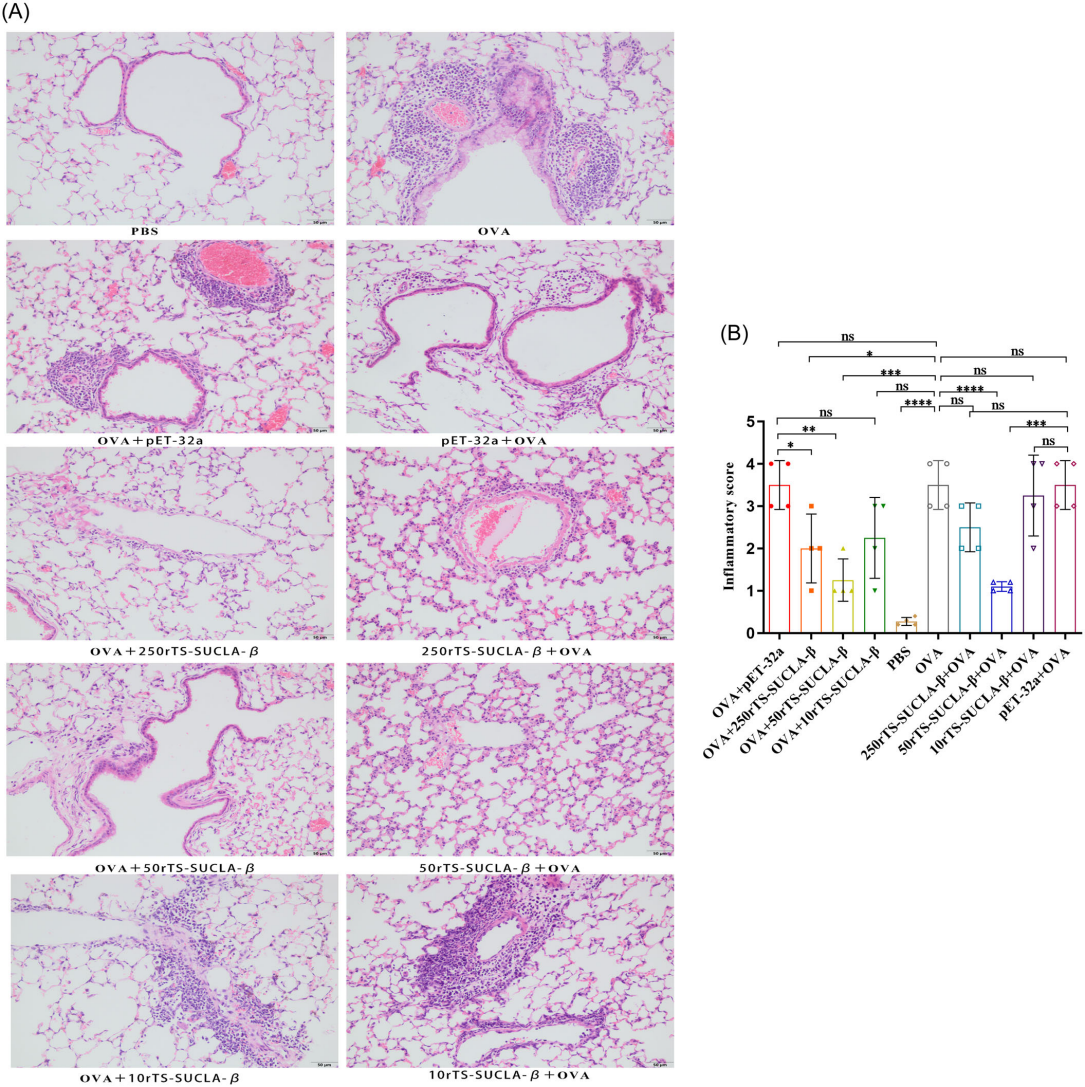
(A): Histopathological changes of pulmonary tissues of OVA‐sensitized/challenged mice with treatment of rTS‐SUCLA‐β in preventive or therapeutic regimens (magnification:200×, Scale bars:50 µm). (B): The improved inflammatory score of lung tissues with treatment of rTS‐SUCLA‐β in preventive or therapeutic regimens (*n* = 5 mice per group, pET‐32a group 50 μg per mouse). *p < .05, **p < .01, ***p < .001, ****p < .0001.

The histochemical observation of lungs in mice showed that PBS and 50rTS‐SUCLA‐β + OVA group, the pulmonary tissue structure was normal, and no eosinophil infiltration was observed in bronchi, perivascular and alveolar septum; OVA, pET‐32a +OVA and OVA+ pET‐32a group had mild focal eosinophil infiltration around pulmonary bronchi, mild or moderate multi‐focal eosinophil infiltration around pulmonary vessels, and no eosinophil infiltration in alveolar septum; 250rTS‐SUCLA‐β + OVA, 10rTS‐SUCLA‐β + OVA, OVA+250rTS‐SUCLA‐β and OVA+50rTS‐SUCLA‐β group, there was slight focal eosinophil infiltration around bronchi and blood vessels. However, there was no eosinophil infiltration in the alveolar septum; OVA+50rTS‐SUCLA‐β group had slight focal eosinophil infiltration around the bronchi, and no eosinophil infiltration around the blood vessels or in the alveolar septa (Figure 3A). As shown in Figure 3B, the inflammation score of the PBS control group was significantly lower than that of OVA, pET‐32a +OVA and OVA+ pET‐32a groups. The scores of OVA+250rTS‐SUCLA‐β, 50rTS‐SUCLA‐β + OVA and OVA+50rTS‐SUCLA‐β groups were significantly lower than those of OVA group, but higher than those of PBS group.

### 3.3 Treatment with rTS‐SUCLA‐β affects the number of eosinophils, lymphocytes, neutrophils and macrophages in BALF

**Figure 4:**
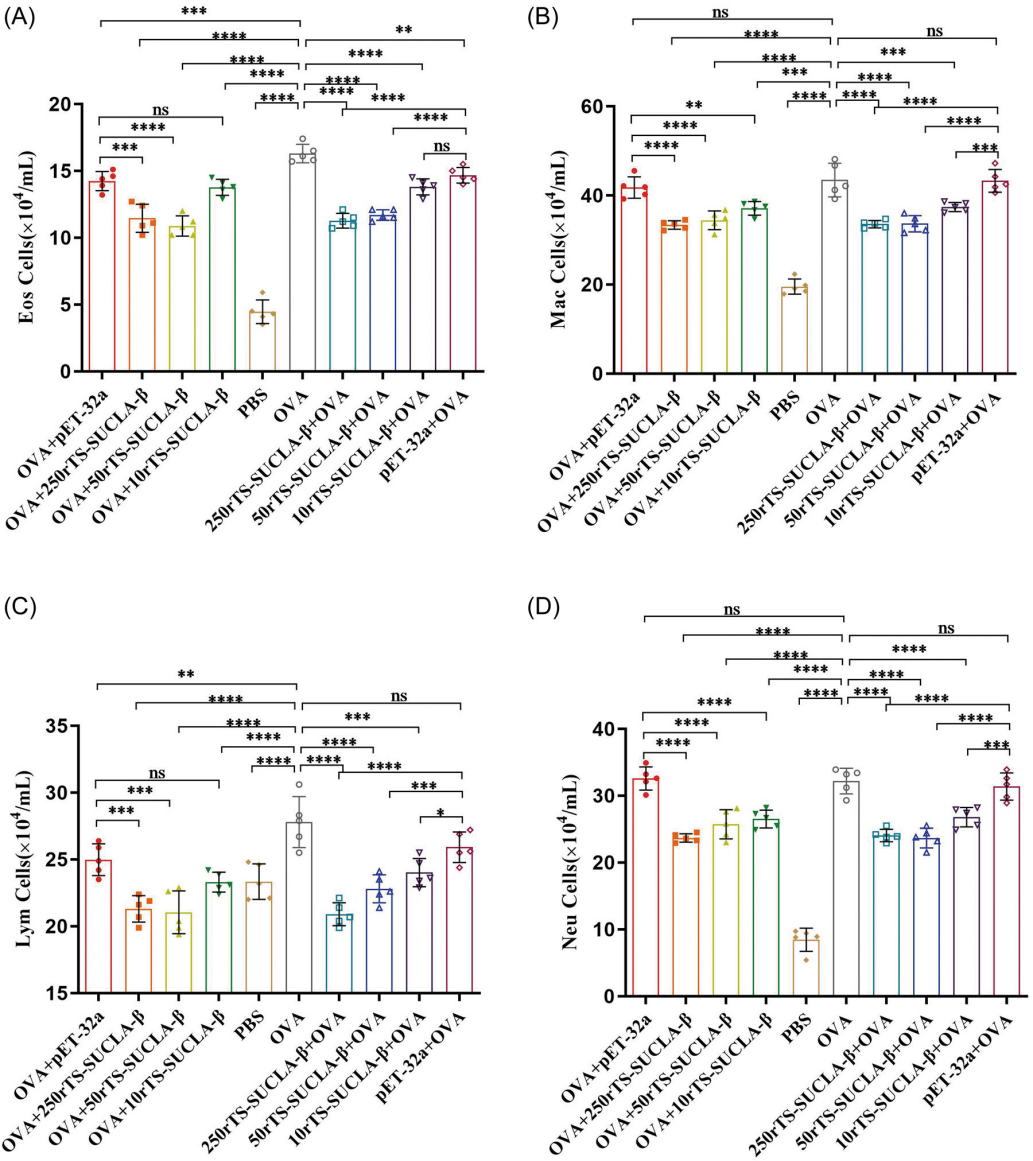
The Eosinophils (A), macrophages (B), lymphocytes (C) and neutrophils (D) cells count in BALF from mice treated with rTS‐SUCLA‐β (*n* = 5 mice per group). *p < .05, **p < .01, ***p < .001, ****p < .0001.

The Eosinophils (Figure 4A) present in BALF of mice that received 250 μg and 50 μg recombinant rTS‐Sucla ‐β showed a significant decrease when compared to those in the OVA and pET‐32a groups. Additionally, the levels of lymphocytes (Figure 4C) were found to be significantly reduced in the groups treated with OVA+250rTS‐SUCLA‐β, OVA+50rTS‐SUCLA‐β, 250rTS‐SUCLA‐β + OVA, 50rTS‐SUCLA‐β + OVA and 10rTS‐SUCLA‐β + OVA, as compared to those treated with OVA and pET‐32a. Furthermore, both macrophages (Figure 4B) and neutrophils (Figure 4D) observed in BALF of mice administered with any dose of recombinant protein exhibited a significant reduction as compared to those seen in the OVA and pET‐32a groups.

### 3.4 Treatment with rTS‐SUCLA‐β affects the secretion of cytokines in BALF

**Figure 5:**
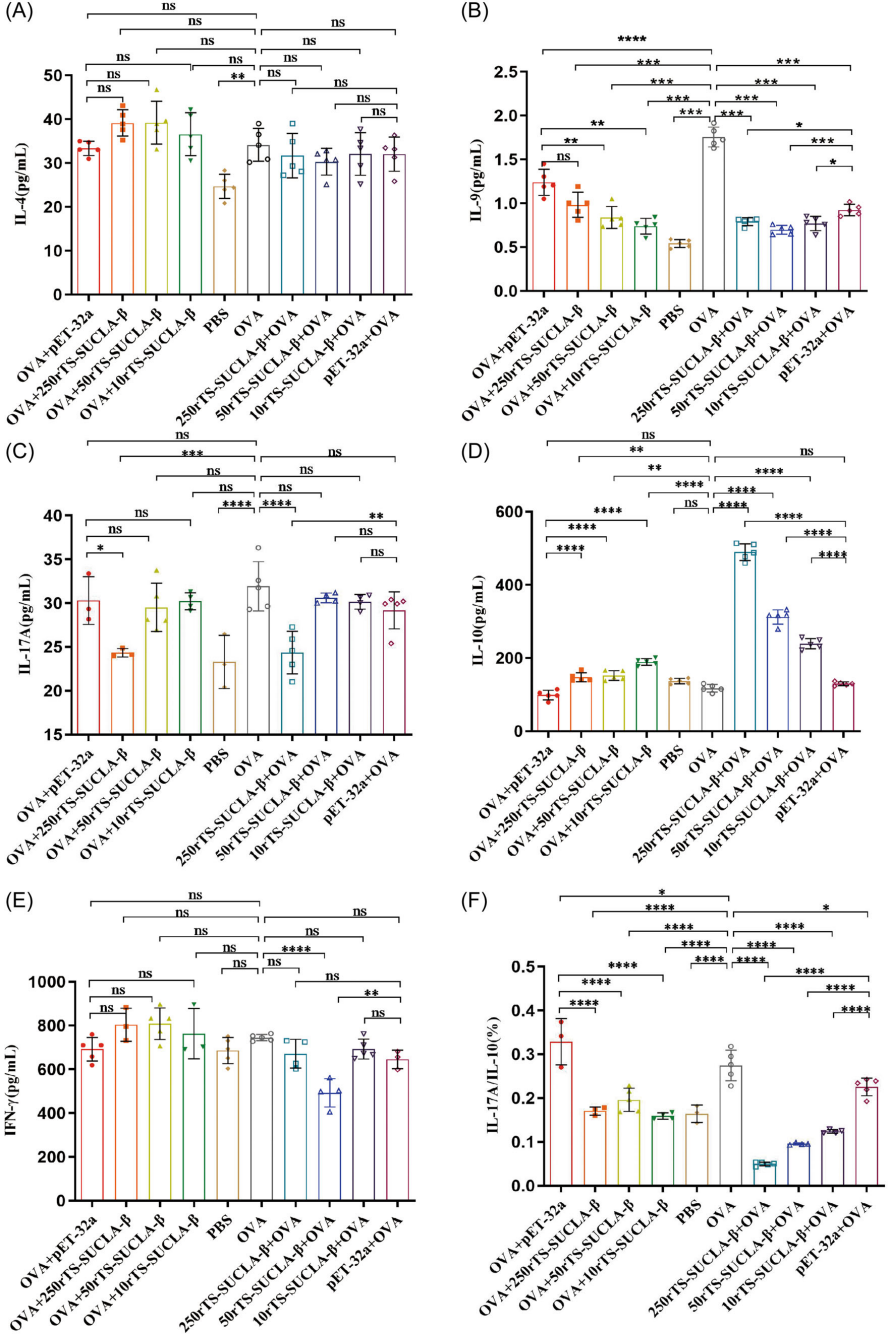
The cytokine levels of IL‐4 (A), IL‐9 (B), IL‐17A (C), IL‐10 (D) and IFN‐γ (E) were measured in BALF of OVA‐sensitized mice upon the treatment of rTS‐SUCLA‐β in preventive and therapeutic models (*n* = 5 mice per group), the ratios of IL‐17A and IL‐10 are illustrated by F. *p < .05, **p < .01, ***p < .001, ****p < .0001.

ELISA kits were used to detect the contents of IL‐4 (Figure 5A), IL‐9 (Figure 5B), IL‐17A (Figure 5C), IL‐10 (Figure 5D) and IFN‐γ (Figure 5E) in BALF. The analytical contents of IFN‐γ and IL‐10 in BALF of OVA injection group were not significantly different from those in PBS group, IFN‐γ secretion in BALF of mice injected with 50rTS‐SUCLA‐β + OVA was significantly lower than that in OVA and pET‐32a+OVA groups. The secretion of IL‐10 in BALF in OVA+250rTS‐SUCLA‐β, OVA+50rTS‐SUCLA‐β, OVA+10rTS‐SUCLA‐β, 250rTS‐SUCLA‐β + OVA, 50rTS‐SUCLA‐β + OVA and 10rTS‐SUCLA‐β + OVA groups was significantly higher than that in OVA and pET‐32a groups. The secretion of IL‐4, IL‐9 and IL‐17A in BALF of OVA injection group was significantly higher than that of PBS control group. The concentration of IL‐9 in BALF of mice injected with rTS‐SUCLA‐β before OVA sensitization was significantly lower than that in OVA group and pET‐32a+OVA group. Receiving an injection of rTS‐SUCLA‐β following OVA sensitization resulted in a dose‐dependent decrease in the secretion of IL‐9 in the BALF of mice, compared to the OVA group. The concentration of IL‐17A in BALF in OVA+250rTS‐SUCLA‐β and 250rTS‐SUCLA‐β + OVA groups was significantly lower than that in OVA and pET‐32a control groups, the ratios of IL‐17A and IL‐10 are illustrated by Figure 5F. IL‐4 were not affected by different doses of recombinant protein injected before and after OVA induced allergic asthma. The findings suggest that the Ts‐SUCLA‐β protein derived from *T. spiralis* triggered immune responses that led to the production of regulatory cytokines such as IFN‐γ, IL‐4, IL‐9, IL‐10, and IL‐17A.

### 3.5 Treatment with rTS‐SUCLA‐β reduced OVA‑specific IgE level in OVA sensitized mice

**Figure 6:**
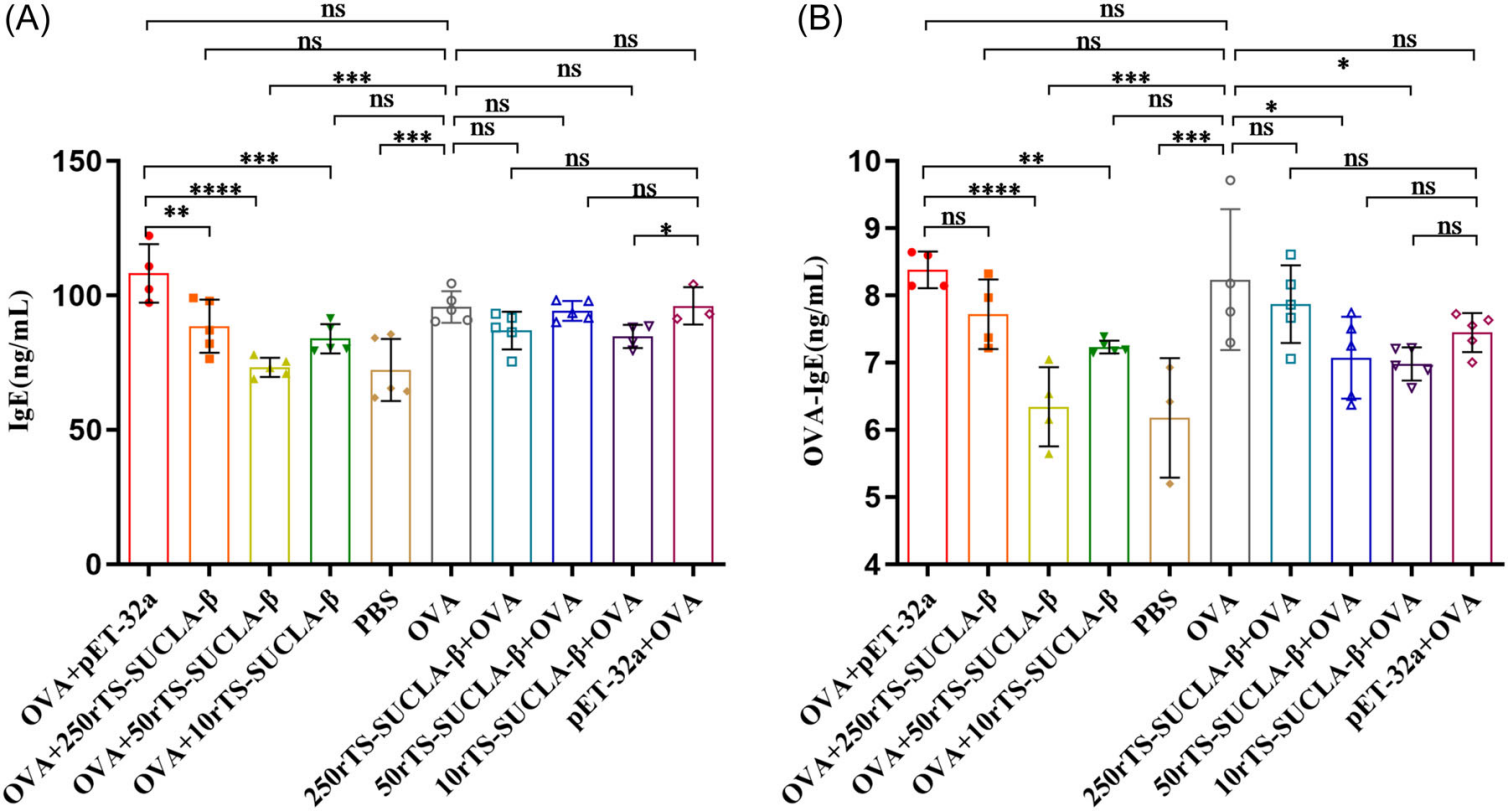
The levels of total IgE (A) and OVA‐specific IgE (B) were measured by ELISA in sera of OVA‐sensitized mice treated with rTS‐SUCLA‐β in preventive and therapeutic models (*n* = 5 mice per group). *p < .05, **p < .01, ***p < .001, ****p < .0001.

The serum levels of total IgE and OVA‐specific IgE exhibited a notable elevation in mice that underwent sensitization with OVA. In the preventive models, administration of rTS‐SUCLA‐β did not result in any notable change in the total serum IgE levels; however, serum total IgE levels (Figure 6A) in OVA+50rTS‐SUCLA‐β group was significantly lower than those in OVA and OVA+pET‐32a groups. Serum OVA‐specific IgE levels (Figure 6B) in OVA+50rTS‐SUCLA‐β group was significantly lower than that in OVA and OVA+pET‐32a groups; serum OVA‐specific IgE level in 50rTS‐SUCLA‐β + OVA and 10rTS‐SUCLA‐β + OVA groups were significantly lower than those in the OVA group, but the difference was not statistically significant compared with pET‐32a+OVA. This suggests that prior administration of rTS‐SUCLA‐β may lead to a decrease in the mouse's IgE level following OVA sensitization/challenge.

### 3.6 rTS‐SUCLA‐β protein regulates the differentiation of Th17 and treg cells in OVA sensitized mice

**Figure 7:**
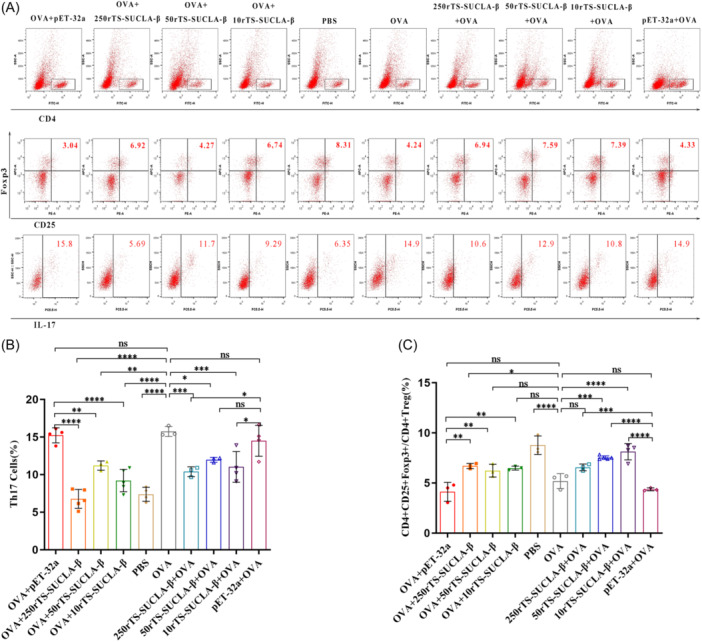
The differentiation of Th17 and Treg cells were measured by flow cytometry in spleen cells of OVA‐sensitized mice treated with rTS‐SUCLA‐β in preventive and therapeutic models (*n* = 5 mice per group). (A): Dot plots analysis and proportion of spleen cells derived‐Th17 (gated on CD4+ and IL‐17A) and Treg (gated on CD4 + CD25+ and Foxp3) cells; (B): Percentage of differentiation of Th17 cells; (C): Percentage of differentiation of Treg cells. *p < .05, **p < .01, ***p < .001, ****p < .0001.

To evaluate the influence of administering rTS‐SUCLA‐β treatment on the differentiation of Treg and Th17 cells, we employed FACS analysis to measure the subsets of CD4+ T cells isolated from mouse spleens (Figure 7A). Compared to the PBS control group, a remarkable decrease in Treg cell differentiation and a significant elevation in Th17 cell differentiation were observed in the OVA group. Th17 cells (Figure 7B) in spleen lymphocytes of mice in OVA+ 250rTS‐SUCLA‐β, OVA+ 50rTS‐SUCLA‐β, 250rTS‐SUCLA‐β + OVA and 10rTS‐SUCLA‐β + OVA groups were significantly lower than those in OVA group and pET‐32a control group. Treg cells (Figure 7C) in spleen lymphocytes in OVA+ 250rTS‐SUCLA‐β, 50rTS‐SUCLA‐β + OVA and 10rTS‐SUCLA‐β + OVA groups were significantly higher than those in OVA group and pET‐32a control group. The findings above confirmed that rTS‐SUCLA‐β treatment regulates host immunity by promoting Treg cells differentiation and inhibiting Th17 cells differentiation.

## 4 DISCUSSION

Asthma is distinguished by the existence of persistent inflammation in the airways, characterized by the infiltration of different types of inflammatory cells like eosinophils, T cells, mast cells, neutrophils, and dendritic cells into the submucosa of the respiratory system.^1^, ^5^ This persistent infiltration results in sustained and long‐term inflammation within the airways.^23^ To assess the potential of rTS‐SUCLA‐β as a viable alternative for treating worm infections and alleviating inflammatory reactions associated with asthma, we administered SUCLA‐β derived from *T. spiralis* ML to an OVA‐induced asthma mouse model either before OVA sensitization (preventive) or during sensitization (therapeutic). The results demonstrated that the administration of 50 μg rTS‐SUCLA‐β to mice before OVA sensitization (as a prophylactic measure) significantly reduced the presence of inflammatory cells surrounding the airway and blood vessels in OVA‐sensitized mice following exposure to OVA. The findings obtained from this study are consistent with those reported in a previous investigation.^18^ In addition, we found that the pathological scores of the treated mice in the 250 ug rTS‐SUCLA‐β group were higher than those in the 50ug rTS‐SUCLA‐β group, and both of these groups were significantly lower than those in the OVA group, however, there was no significant change in the 10ug rTS‐SUCLA‐β group. These results suggest that treatment with too low concentration of rTS‐SUCLA‐β may not affect the allergic inflammatory lesions, whereas treatment with a high concentration of rTS‐SUCLA‐β may increase the effect of pulmonary inflammatory lesions Therefore, it is particularly important to choose the optimal concentration.^24^

Allergic asthma is commonly identified by increased Th2 cytokine levels and serum IgE concentrations.^9^ The levels of eosinophils in the BALF and OVA‐specific IgE in the serum were significantly reduced in both the therapeutic and preventive groups compared to the OVA group. These findings indicate that rTS‐SUCLA‐β has potential for alleviating the pathological symptoms associated with OVA‐induced allergic asthma in mice. The immune response in asthma primarily involves the activation of Th2 cells, which subsequently migrate to the airways and induce an upregulation of Th2‐type cytokines.^2^ Th1 cells possess the capability to suppress the development of Th2 cells and exert anti‐inflammatory effects in individuals with asthma.^25^ Nevertheless, an upsurge in Th1 cells may not always be advantageous for allergic asthma as excessive secretion of IFN‐γ by these cells can also instigate airway inflammation.^26^ In this investigation, we have shown that the levels of IFN‐γ in the BALF of mice in the OVA group were comparable to those in the PBS group. However, there was a significant increase in IL‐4 levels compared to the PBS group. rTS‐SUCLA‐β was administered at varying dosages to mice with asthma induced by OVA, and it was showed that IFN‐γ in BALF of 50 μg rTS‐SUCLA‐β + OVA group was decreased, while IL‐4 level in the groups treated with rTS‐SUCLA‐β had no significant change compared with OVA group. This outcome suggests that the therapeutic effect of rTS‐SUCLA‐β in the asthma model may not be mediated by Th1/Th2 responses. Furthermore, another experiment yielded similar results, indicating that helminths impede asthma development through the secretion of substances that modulate allergic reactions, while leaving helminth‐specific Th2 immune response generation unaffected.^27^

The cytokine IL‐10 has a unique function in safeguarding against allergic airway inflammation in individuals with asthma.^5^ The presence of this particular cytokine plays a crucial role in the management of Trichinellosis‐related illness, aiding in the survival of the host while also suppressing the immune response towards the parasite.^28^, ^29^ Treatment with rTS‐SUCLA‐β, similar to other helminth infections, induces an elevation in IL‐10 levels, indicating that the immunomodulatory effects of rTS‐SUCLA‐β are mediated through activation of the regulatory pathway within the immune system. The presence of IL‐10 plays a pivotal role in suppressing asthma symptoms by modulating the activity of APC and attenuating IgE production.^5^ In addition to the presence of helminth infection, exposure to helminth extracts or products can also lead to an increased level of IL‐10.^13^ The observed rise in IL‐10 levels after administering rTS‐SUCLA‐β treatment in mice sensitized with OVA is likely a result of Treg activation induced by this therapy, which corresponds with the upregulation of Treg cells identified through flow cytometry analysis in mice with OVA‐induced asthma as reported in this study. It is widely acknowledged that Tregs have a significant impact on the immune system's regulation during helminth infections in their hosts.^30^, ^31^ Treg production is induced by infection with *T. spiralis* or other helminths or by helminth‐derived proteins and can alleviate asthma and other inflammatory diseases,^32^, ^33^, ^34^ indicating its ability to reduce hypersensitivity towards allergens or autoantigens. These findings emphasize the possibility of utilizing helminth‐derived proteins for the treatment or prevention of allergic or autoimmune inflammations, offering valuable insights into their mechanisms of action.

The maintenance of Th1/Th2 balance is essential for preventing the onset of asthma.^35^ Recent research has highlighted the significant role played by Th17/Tregs in the intricate processes associated with the advancement and development of asthma.^36^ In individuals with moderate to severe asthma, an upregulation of IL‐17A levels was observed in Th17 cells, as well as in the plasma and culture supernatant.^35^ In this research, there was a notable increase in the differentiation of Th17 cells in spleen lymphocytes and secretion of IL‐17A in BALF when compared to the control group treated with PBS. IL‐17A, acknowledged as the proinflammatory component of tissues, has been recognized as a pivotal cytokine in combating parasitic infections and demonstrating diverse functionalities.^37^ Th17 cells play a role in the synthesis of IL‐17A and control of inflammatory responses, thus enhancing our comprehension of immune reactions.^38^ Previous studies have demonstrated the potential of ES‐62 derived from *A. viteae* and Cystatins for *A. cantonensis* to ameliorate the asthmatic response by reducing IL‐17A levels.^39^, ^40^, ^41^, ^42^, ^43^ Our research revealed that administering rTS‐SUCLA‐β led to an increase in Treg cells and a decrease in Th17 cells, resulting in reduced expression of IL‐17A when compared to the OVA group. These results indicate that rTS‐SUCLA‐β has the ability to regulate host immunity and play a significant role in managing allergic asthma.

In the past few years, researchers have identified a unique subset of CD4+ T cells called T helper 9 cells. These particular cells are distinguished by their ability to produce IL‐9, which is regarded as a crucial cytokine. Surprisingly, T helper 9 cells have been discovered to be involved in tumor defense mechanisms, allergic responses, and the manifestation of asthma indications.^44^, ^45^ Repression of IL‐9 synthesis can be accomplished by utilizing a secretory substance derived from parasites, thus providing potential safeguard against experimentally induced asthma.^45^ This research, we have demonstrated that treatment with rTS‐SUCLA‐βeffectively mitigates the allergen‐specific Th9 response, particularly by significantly reducing the levels of IL‐9 in BALF. This reduction holds potential for exerting a protective effect on OVA‐induced asthma inflammation; however, further experimental investigations are warranted to ascertain the precise underlying mechanism of this protection. Similar outcomes were observed in the pET‐32a control group as in the rTS‐SUCLA‐β treatment group. In our previous investigation, we observed an elevation in the serum level of IL‐9 antibody in *T. spiralis* infected mice following pET‐32a treatment; however, this increase was not statistically significant when compared to the blank control group.^20^ The findings of this study imply that pET‐32a potentially modulates the secretion of IL‐9; however, further investigation is warranted to elucidate the precise regulatory mechanism in future studies. Our findings clearly demonstrate that the rTS‐SUCLA‐β protein obtained from *T. spiralis*, has the ability to alleviate airway inflammation in a mouse model of OVA‐induced asthma by facilitating the activation of the host immune system. Additionally, another experiment yielded a comparable outcome indicating that mangiferin demonstrated anti‐asthmatic properties by reducing the Th9 and Th17 responses while enhancing the Treg response in a mouse model of OVA‐induced asthma.^46^ Further research on the mechanism by which rTS‐SUCLA‐β regulates the host's immune response is imperative. This will significantly contribute to optimizing therapeutic efficacy, ensuring safety, and facilitating large‐scale production of recombinant proteins as innovative therapeutic agents for the management of allergic and autoimmune inflammatory disorders.

## 5 CONCLUSIONS

In summary, the administration of varying doses of rTS‐SUCLA‐β to mice, either before sensitization as a preventive measure or during stimulation as a treatment approach, demonstrates potential for downregulating eosinophil levels surrounding bronchial and vascular areas and decreasing the presence of inflammatory cells in BALF. The levels of proinflammatory cytokines such as IFN‐γ, IL‐17A, and IL‐9 were found to be reduced in BALF, whereas there was an observed increase in the concentration of IL‐10. The balance between Th17 and Treg cells is crucial in regulating the population of inflammatory cells in the body, leading to the improvement of inflammation and relief from symptoms linked to allergic asthma. In the prevention and treatment of allergic asthma in mice, 50 μg rTS‐SUCLA‐β showed the most obvious effect.

## AUTHOR CONTRIBUTIONS

**Kalibixiati Aimulajiang**: Formal analysis; methodology; writing—original draft; writing—review and editing. **Wen Chu**: Conceptualization; data curation; methodology; resources. **Shuyi Liao**: Resources; visualization. **Zhaohai Wen**: Conceptualization; formal analysis. **Rongdong He**: Formal analysis. **Mingmin Lu**: Conceptualization; data curation; resources. **Lixin Xu**: Conceptualization; supervision. **Xiaokai Song**: Software; supervision. **Xiangrui Li**: Supervision. **Ruofeng Yan**: Conceptualization; funding acquisition; resources; supervision; writing—review and editing.

## CONFLICT OF INTEREST STATEMENT

The authors declare no conflict of interest.

## Data Availability

I confirm that my article contains a Data Availability Statement even if no data is available (list of sample statements) unless my article type does not require one.
